# Overexpression of LINC00673 Promotes the Proliferation of Cervical Cancer Cells

**DOI:** 10.3389/fonc.2021.669739

**Published:** 2021-05-21

**Authors:** Sheng-Kai Huang, Ruo-Xuan Ni, Wen-Jie Wang, Di Wang, Mei Zhao, Cheng-Zhi Lei, Xiao-Jie Sun, Chang-Zhi Huang, Ping Bai, Yi-Qun Che, Jian-Ping Xu

**Affiliations:** ^1^ Department of Clinical Laboratory, National Cancer Center/National Clinical Research Center for Cancer/Cancer Hospital, Chinese Academy of Medical Sciences and Peking Union Medical College, Beijing, China; ^2^ Department of Etiology and Carcinogenesis, State Key Laboratory of Molecular Oncology, Beijing Key Laboratory for Carcinogenesis and Cancer Prevention, National Cancer Center/National Clinical Research Center for Cancer/Cancer Hospital, Chinese Academy of Medical Sciences and Peking Union Medical College, Beijing, China; ^3^ The Center for Disease Control and Prevention of Huai Rou, Beijing, China; ^4^ Department of Gynecologic Oncology, National Cancer Center/National Clinical Research Center for Cancer/Cancer Hospital, Chinese Academy of Medical Sciences and Peking Union Medical College, Beijing, China; ^5^ Department of Biochemistry, Qiqihar Medical University, Qiqihar, China; ^6^ Department of Medical Oncology, National Cancer Center/National Clinical Research Center for Cancer/Cancer Hospital, Chinese Academy of Medical Sciences and Peking Union Medical College, Beijing, China

**Keywords:** LINC00673, cervical cancer, proliferation, overexpression, lncRNA

## Abstract

**Objective:**

To study the expression of LINC00673 in cervical cancer and cervical intraepithelial neoplasia (CIN) and to explore the role of LINC00673 in the development of cervical cancer.

**Methods:**

The expression of LINC00673 in serum from cervical cancer patients, CIN patients, and healthy participants was detected by RT-qPCR. The function of LINC00673 in cervical cancer cells was analyzed using *in vitro* and *in vivo* experiments.

**Results:**

Our results revealed that serum LINC00673 levels were highest in cervical cancer patients, followed by patients with CIN and healthy controls. *In vitro* experiments demonstrated that overexpression of LINC00673 enhanced the proliferation and cell cycle progression of HeLa and SiHa cells. *In vivo* experiments showed that the tumor weight and volume of nude mice subcutaneously injected with LINC00673-overexpressing HeLa cells were larger than those of nude mice injected with control cells (*P* < 0.05). Western blotting showed that cell cycle-related proteins cyclin A2 and cyclin E and interstitial-associated proteins Snail and N-cadherin were upregulated and p53 signaling pathway-related proteins were downregulated in LINC00673-overexpressing HeLa and SiHa cells.

**Conclusion:**

LINC00673 plays an important role in the development of cervical cancer and may serve as a new therapeutic target for cervical cancer.

## Introduction

Cervical cancer is the second most common cancer among women worldwide and a major risk factor for women’s health (1). Although the increase in early screening has significantly reduced the mortality rate of patients with cervical cancer, a high proportion of cervical cancer patients are diagnosed in advanced stages (stage III and IV) (2). Therefore, better understanding of the biological mechanisms of cervical cancer with the aim of identifying new diagnostic biomarkers and therapeutic targets is of great significance for patients with this disease.

Long non-coding RNAs (lncRNAs) are RNA transcripts that are longer than 200 nucleotides and lack protein-coding function (3). LncRNAs were once considered as garbage sequences generated during genome evolution because of their inability to encode functional proteins. However, a study in 2007 by Rin (4) described the biological function of the lncRNA HOTAIR (HOXIR, transcript antisense RNA). Since then, a large number of reports have found that lncRNAs play important biological functions, and lncRNAs have a wide range of tissue expression profiles and strong tissue expression specificity. Multiple studies have found that lncRNAs play key roles in tumorigenesis and development, and several lncRNAs are differentially expressed in cancers, including cervical cancer (5–8). For example, the lncRNA LINC00673 has a carcinogenic effect in breast cancer (9, 10), lung cancer (11, 12), and gastric cancer (13) and exerts a tumor suppressive effect in pancreatic cancer (14). The expression of LINC00673 was also studied in cervical cancer. Shi et al. showed that LINC00673 is overexpressed in cervical cancer tissues and is associated with poor prognosis (15). However, other studies showed that LINC00673 expression was lower in cancer tissues compared with adjacent normal tissues, and the LINC00673 rs11655237 single nucleotide polymorphism can be used as a prognostic biomarker of cervical cancer (16, 17). Thus, the biological function of LINC00673 in cervical cancer has been controversial. Therefore, in this study, we examined the role of LINC00673 in the occurrence and development of cervical cancer using *in vitro* and *in vivo* experiments.

## Materials and Methods

### Patients and Ethics Statement

All cervical cancer patients and cervical intraepithelial neoplasia (CIN) patients in this study were treated at the Cancer Hospital, Chinese Academy of Medical Sciences from May 2016 to August 2017. The CIN patients include CIN2 and CIN3. Diagnosis was confirmed by pathology and histology. The participant characteristics are shown in **Table 1**.

**Table 1:** Clinicopathological features of patients included in this study.

Characteristics	Healthy	CIN2	CIN3	Cancer
**Age**				
<48 years	30	8	21	33
≥48 years	41	3	10	36
**Pathological type**				
squamous cell carcinoma	–	–	–	43
adenocarcinoma	–	–	–	26
**FIGO stage**				
I	–	–	–	52
II-IV	–	–	–	17
**Tumor size**				
<3 cm	–	–	–	37
≥3 cm	–	–	–	32
**Lymphatic metastasis**				
N0	–	–	–	57
N1 or above	–	–	–	12

Federation International of Gynecology and Obstetrics (FIGO).

Data are shown as n.

The ethical committee of Cancer Hospital, Chinese Academy of Medical Sciences approved all experimental protocols for using patient samples. All methods were performed according to the relevant guidelines and regulations.

### Serum Collection, RNA Isolation, and RT-qPCR

We collected venous blood samples (<4 mL) from patients using vacuum blood collection tubes. After resting samples at room temperature for 30 min, the samples were centrifuged at 4°C at 820 g for 10 min. The isolated serum was transferred into new 1.5 mL eppendorf tubes, followed by centrifugation at 8,000 g at 4°C for 10 min. Serum samples were transferred to new RNase- and DNase-free 0.5 mL eppendorf tubes and stored at -80°C until total RNA extraction.

Total RNA was isolated from 250 µL of serum using 750 µL of Trizol LS Reagent (Invitrogen, USA). To isolate total RNA from cultured cells, 1 mL of Trizol Reagent (Invitrogen) was used. Next, 200 µL of trichloromethane was added to accelerate the separation of the RNA phase. Isopropanol was used to precipitate total RNA, and samples were washed with 75% ethanol. Finally, 26 µL of RNase-free water was used for solubilization.

We used a reverse transcriptase M-MLV kit (Takara, Japan) to synthesize the first-strand cDNA according to the manufacturer’s protocol. The cDNAs were diluted to appropriate concentrations with RNase-free water and used as templates for subsequent analysis. RT-qPCR was performed using the SYBR Premix Ex Taq II kit (Takara) in a 7500 Real-Time PCR System (Applied Biosystems, USA). Melt curve analyses were used to confirm the specificity of each PCR reaction. GAPDH mRNA served as an internal reference; the expression level of each gene was normalized by the expression level of GAPDH mRNA according to the 2^-ΔΔCT^ method. Each experiment was performed in triplicate and repeated three times. The primer sequences are shown in **Table S1**.

### Cell Culture

We obtained 293T, SiHa, and HeLa cells from the American Type Culture Collection (ATCC) and cultured the cells according to the instructions of ATCC. All cell lines were cultured in DMEM (Hyclone, USA) supplemented with 10% fetal bovine serum (FBS). All cell lines were cultured in a final concentration of 100 U/mL penicillin and 100 mg/mL streptomycin at 37°C with 5% CO_2_.

### Generation of LINC00673 Overexpression and Control Cell Lines

The LINC00673 sequences were commercially synthesized by Sangon Biotech (China) and inserted into a pLVX-Puro lentiviral expression vector. The empty pLVX-Puro lentiviral expression vector served as the control vector. Lentivirus particles were packaged by co-transfecting packaging plasmids (pMD2.G and psPAX2) with pLVX-Puro lentiviral expression vectors into 293T cells using PEI transfection reagent. We collected virus at 48 h and 72 h after transfection. HeLa and SiHa cells were then infected using LINC00673 and control lentivirus particles, and stable cell lines were selected for 48 h in culture medium containing 2 μg/mL puromycin. The packaging and infection of lentivirus were all carried out in the biosafety cabinet.

### Cell Proliferation Assay

HeLa and SiHa cells were seeded in 96-well plates (1000 cells in 100 μL per well and 3000 cells in 100 μL per well, respectively). Cell survival was measured daily over 6 days using MTT assays. Briefly, 90 μL culture medium combined with 20 μL of MTT solution (Sigma, USA) was added to cells at specific time points. After 4 h, the medium was replaced with 150 μL of dimethyl sulfoxide. The OD was measured at 492 nm on a microplate spectrophotometer. All experiments with six replicates were assessed in triplicate.

### Colony Formation Assay

Cells were plated into 6-well plates at a density of 150 cells per well and cultured in complete medium containing 10% FBS. Cells were cultured for 1–2 weeks until visible clones formed. Cell colonies were incubated with methanol for 15 min and Giemsa solution was added for 15 min to stain cells. The stained colonies were counted and imaged. Each well was repeated three times.

### Cell Cycle Assay

Cells were harvested and fixed in 70% ethanol at 4°C overnight. Single-cell suspensions were then stained with 50 μg/mL propidium iodide (Sigma) at room temperature for 30 min and analyzed by flow cytometry (BD LSR II, USA).

### *In Vivo* Assays

Female BALB/c nude mice (4-week-old) were obtained from Beijing Huafukang Bioscience Corporation. Mice were adapted to the new conditions for 7 days and raised under a 12-h dark/12-h light cycle with sufficient food and water. HeLa cells were infected with pLVX-Puro LINC00673 lentivirus particles or empty vector lentivirus particles and resuspended at a concentration of 1×10^7^ cells/mL. Mice (n=5 each group) were subcutaneously injected with cell suspensions (1 × 10^6^ cells in 100 μL PBS) in the dorsal right flank. At 25 days post-injection, the mice were sacrificed; the tumors were removed, photographed, and measured. All procedures involving the use of animals were performed according to the Institutional Animal Care and national guidelines.

### Western Blot

Samples were lysed in 1× lysis buffer (150 mM NaCl, 1.5% NP-40, 50 mM Tris-HCl, pH 7.4, 0.1% sodium dodecyl sulfate [SDS]). Protein samples were separated by sodium dodecyl sulfate polyacrylamide gel electrophoresis (SDS-PAGE) and then transferred onto polyvinylidene fluoride (PVDF) membranes. The membranes were incubated with the following primary antibodies at 4°C overnight: GAPDH (1:1000, Cell Signaling Technology, USA); cyclin A2 (1:1000, Cell Signaling Technology); Snail(1:1000, Cell Signaling Technology); N-cadherin(1:1000, Cell Signaling Technology); cyclin E (1:1000, Cell Signaling Technology); AKT (1:1000, Cell Signaling Technology); p-AKT (1:1000, Cell Signaling Technology); p53 (1:1000, Santa Cruz, USA); and p21 (1:1000, Abcam, UK). Membranes were then incubated with goat anti-rabbit or anti-mouse secondary antibodies for 1 h at room temperature. ECL detection reagent was used to detect peroxidase-conjugated secondary antibody. We used GAPDH as the loading control.

### Statistical Analysis

Statistical analysis was performed using SPSS (Statistical Package for Social Sciences, version 22.0) and GraphPad Prism (version 6.01). Student’s t-test, and nonparametric test. All *P*-values were two sided and *P*<0.05 was considered statistically significant.

## Results

### The Expression of LINC00673 in the Serum of Cervical Cancer Was Higher Than in CIN and Healthy Controls

**Figure 1:**
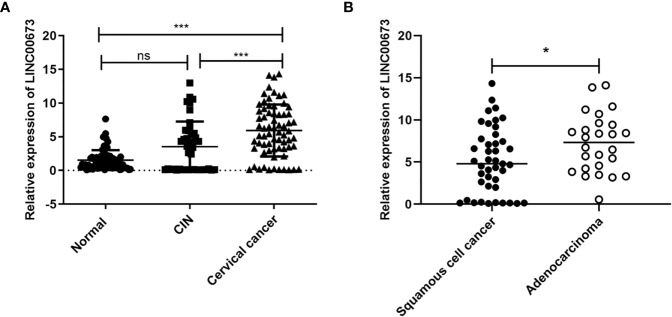
LINC00673 is expressed at different levels in different populations. **(A)** RT-qPCR shows that the level of LINC00673 was gradually increased in serum from healthy controls (n = 71), CIN patients (n = 42), and cervical cancer patients (n = 69). **(B)** LINC00673 in serum was significantly lower in squamous cell carcinoma patients (n = 43) than in adenocarcinoma patients (n = 26). *P < 0.05, ***P < 0.001. Student’s *t*-test. ns, not statistically significant.

We examined the expression of LINC00673 in peripheral blood samples from 69 patients with cervical cancer, 42 patients with CIN2/3, and 71 healthy controls. Our results showed that serum LINC00673 levels were higher in cervical cancer patients than CIN patients and healthy controls (*P* < 0.001) (**Figure 1A**).

Correlation analysis between serum LINC00673 expression level and clinicopathological characteristics of tumors showed that serum LINC00673 expression was significantly associated with the tumor pathological type (*P* < 0.05) (**Figure 1B**).

### Overexpression of LINC00673 Promoted Cell Proliferation of HeLa and SiHa Cells

**Figure 2:**
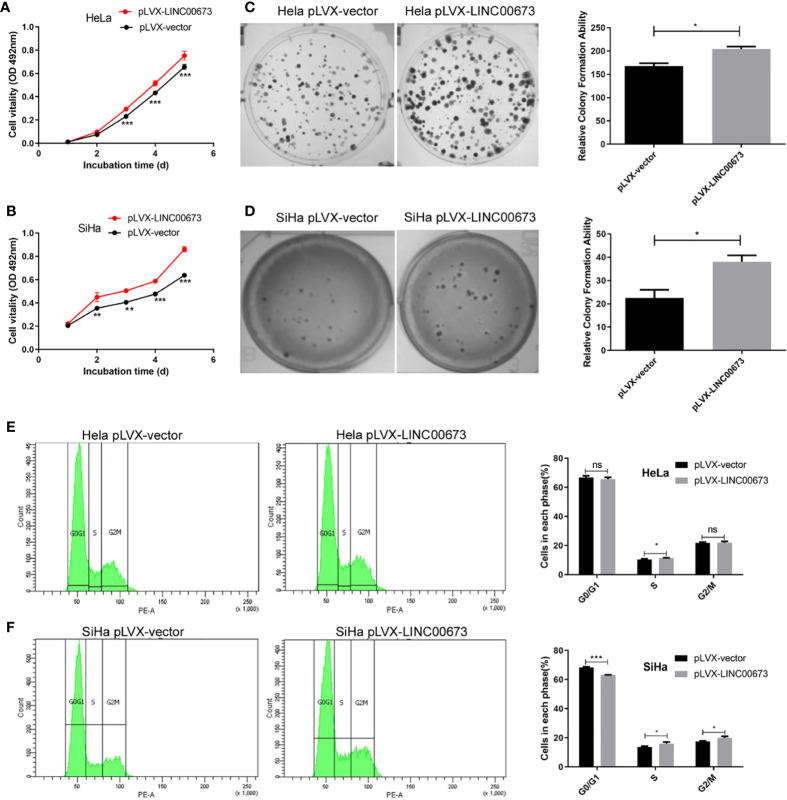
LINC00673 promotes the proliferation, colony formation and cell cycle of cervical cancer cells. Experiments were performed in stable cell lines with lentivirus-mediated overexpression of LINC00673 or controls. **(A, B)** MTT proliferation assay indicated that LINC00673 increases cell proliferation in both HeLa cells and SiHa cells. **(C, D)** LINC00673 promotes the colony formation ability of HeLa and SiHa cells. The bar chart shows the corresponding colony formation number. **(E, F)** Cell cycle distributions of **(E)** HeLa cells and **(F)** SiHa cells overexpressing LINC00673 and control cells were examined using flow cytometry. Percentages of cells in each phase are indicated. Data are presented as mean ± SD (N = 3); *P < 0.05, **P < 0.01, ***P < 0.001. Student’s *t*-test. ns, not statistically significant.

We overexpressed LINC00673 in HeLa and SiHa cells using lentivirus and confirmed LINC00673 overexpression by RT-qPCR (**Figures S1A, B**). We found that overexpression of LINC00673 enhanced the proliferation of HeLa and SiHa cells (**Figures 2A, B**). Colony formation assays revealed that the colony formation ability of HeLa and SiHa cells with LINC00673 overexpression was increased compared with controls (*P*<0.05) (**Figures 2C, D**).

### Overexpression of LINC00673 Promoted Cell Cycle Progression of HeLa and SiHa Cells

To investigate the enhanced cell proliferation by LINC00673 in more detail, we used flow cytometry to examine cell cycle distributions in HeLa and SiHa cells overexpressing LINC00673 compared with control cells. Overexpression of LINC00673 in SiHa cells caused a decrease in G0/G1 phase cells compared with controls (68.3 ± 0.51% vs. 63.1 ± 0.25%, *P* < 0.001), and it showed no difference in HeLa cells (66.8 ± 1.17% vs. 65.7 ± 1.25%, *P* = 0.34). Overexpression of LINC00673 in SiHa cells caused an increase in G2/M phase cells compared with controls (17.5 ± 0.36% vs. 20.0 ± 0.93% *P* = 0.012), and it showed no difference in HeLa cells (21.7 ± 0.72% vs. 21.8 ± 1.05%, *P* = 0.86). Overexpression of LINC00673 in cervical cancer cell lines caused an increase in S phase cells compared with controls (10.3 ± 0.57% vs. 11.4 ± 0.26% in HeLa cells (*P* = 0.042) and 13.6 ± 0.64% vs. 16.0 ± 1.01% in SiHa cells (*P* = 0.023), respectively). These results indicate that overexpression of LINC00673 promoted cell cycle progression of HeLa and SiHa cells (**Figures 2E, F**).

### Overexpression of LINC00673 Enhanced Xenograft Tumor Growth in Nude Mice

**Figure 3:**
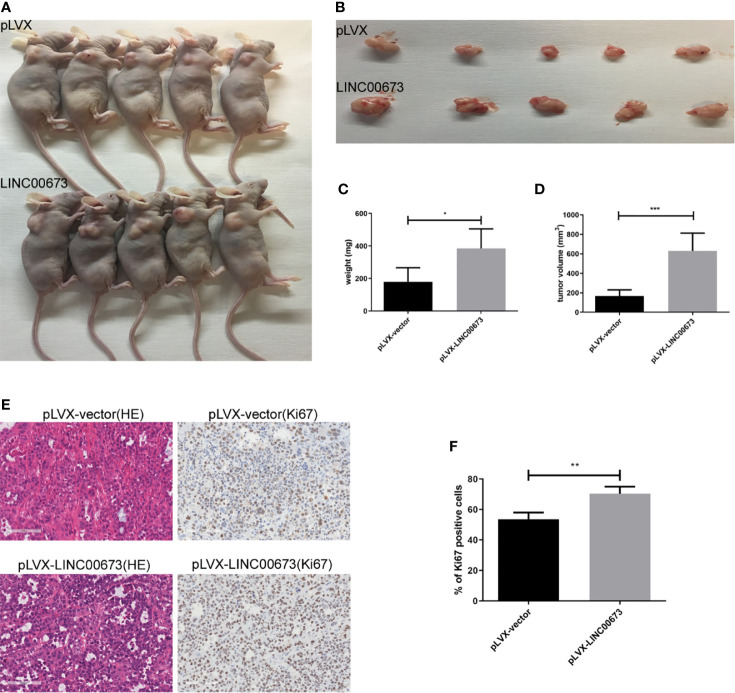
LINC00673 promotes HeLa cell growth *in vivo*. **(A)** HeLa cells overexpressing LINC00673 and control cells were injected into nude mice as described in Methods. Xenograft tumor growth was monitored. **(B–D)** The xenograft tumors were dissected and weighed. **(E)** HE and Ki67 immunohistochemical stained sections from the xenograft tumors. **(F)** The percentages of Ki67 positive cells are indicated. Data are presented as mean ± SD (N = 5); *P < 0.05, **P < 0.01, ***P < 0.001. Student’s *t*-test.

Our *in vitro* experiments showed that overexpression of LINC00673 enhanced cell proliferation by promoting cell cycle progression. To further observe the role of LINC00673 on cervical cancer cells *in vivo*, we conducted tumorigenic experiments in nude mice using HeLa cervical cancer cells infected with LINC00673 or control lentivirus. In the control group injected with control cells, the tumor volume was 167.6 ± 28.01 mm^3^ and the tumor weight was 179.6 ± 38.69 mg. In mice injected with cells with LINC00673 lentivirus, the tumor volume was 630.5 ± 81.4 mm^3^ and the tumor weight was 385.9 ± 53.61 mg, and both tumor volume and weight were significantly greater in the LINC00673 group than the control group (*P* < 0.001 and *P* = 0.0142) (**Figures 3A–D**). This proliferation was also verified by ki67 immunohistochemical staining in the xenograft tumors. The percentages of Ki67 positive cells were 53.5 ± 4.48% and 70.4 ± 4.65% respectively (*P* = 0.020) (**Figures 3E, F**). These results show that LINC00673 promoted the tumorigenicity of the HeLa cervical cancer cell line in nude mice.

### LINC00673 Increases the Expression of IL-17A

**Figure 4:**
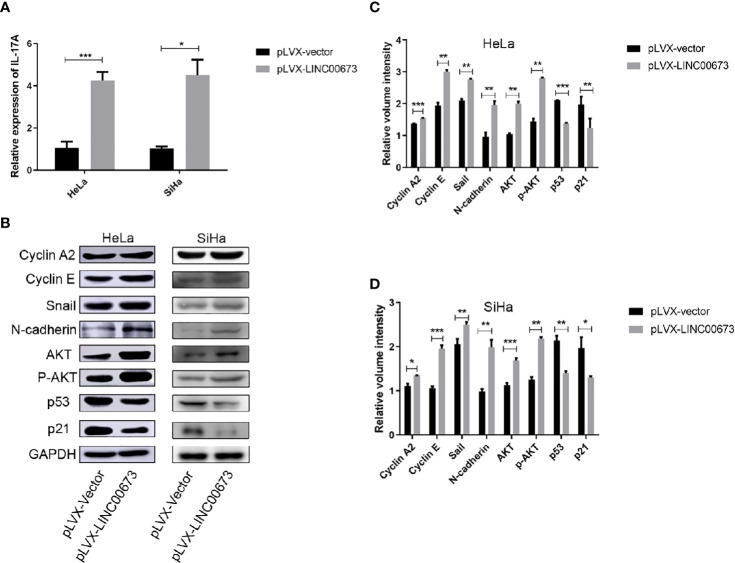
LINC00673 induces IL-17A mRNA and activates AKT-related proteins. **(A)** RT-qPCR results showed that LINC00673 overexpression promoted IL-17A mRNA expression in HeLa and SiHa stable cell lines. **(B)** Detection of AKT-related proteins in LINC00673-overexpressing and control HeLa and SiHa stable cell lines by western blot. GAPDH was used as the loading control. **(C, D)** Bar charts show quantification of the western blot results in **(B)**. Data are presented as mean ± SD (N = 3); *P < 0.05, **P < 0.01, ***P < 0.001. Student’s *t*-test.

Studies have shown that cervical cancers show high levels of inflammatory factors (18). Therefore, we speculated that LINC00673 may exert its biological function by regulating inflammatory factors. We used RT-qPCR to screen mRNA levels of a variety of inflammatory factors closely related to the development of cancer, including IL-6, IL-10, IL-17A, and Hsp90α, in LINC00673 and control stable cells. The results showed that the expression levels of IL-17A mRNA were significantly increased in HeLa and SiHa cells with LINC00673 overexpression compared with control cells (*P <* 0.001, *P* = 0.011, respectively) (**Figure 4A**). In contrast, there were no significant differences in the mRNA expressions of IL-6, IL-10, and Hsp90α in LINC00673-overexpressing cells compared with controls (**Figures S2A, B**).

### Detection of the Tumor Development-Related Signaling Pathways in LINC00673-Overexpressing HeLa and SiHa Cells

We next examined tumor development-related signaling pathway proteins in LINC00673-overexpressing cells and control cells by western blot. Compared with the control cells, LINC00673-overexpressing cells showed increased expression levels of cyclin A2 and cyclin E. The expressions of stromal cell markers Snail and N-cadherin were also increased in LINC00673-overexpressing cells, suggesting that LINC00673 may promote the transformation of tumor cells from epithelial cells to mesenchymal cells. In addition, both AKT total protein and phosphorylated levels were elevated, and protein levels of p53 and p21 were significantly decreased in LINC00673-overexpressing cells compared with controls (**Figures 4B–D**).

## Discussion

Many studies have found that LINC00673 exhibits different biological roles in non-small cell lung cancer, tongue cancer, gastric cancer, and pancreatic cancer. However, the biological function of LINC00673 in cervical cancer has remained controversial. In this study, we analyzed the expression of LINC00673 in peripheral blood serum with the aim of investigating LINC00673 as a potential molecular marker for cervical cancer. The expression of LINC00673 in serum was significantly increased in cervical cancer compared with serum of healthy controls. Large population-level studies have shown that the incidence and mortality rates of cervical cancer have decreased with increased detection and treatment of high-grade cervical histological abnormalities (commonly defined as CIN2+) (18). Therefore, we included patients with CIN2/3 in our study. Although the expression of LINC00673 in CIN2/3 patients was not statistically different from that in healthy controls, we still observed elevated LINC00673 expression in some patients.

We constructed stably infected cervical cancer cell lines overexpressing LINC00673. Overexpression of LINC00673 increased the cell viability of HeLa and SiHa. LINC00673 overexpression in cervical cancer cells also promoted the growth of xenograft tumors in nude mice. These results show that LINC00673 may play an oncogenic function in cervical cancer.

HPV leads to CIN, which can eventually develop to cervical cancer. The presence of inflammatory factors maintains and promotes tumor progression (18). The level of serum IFNβ, IL-1β, and IL-6 was significantly higher in HPV-positive patients compared to healthy controls (19). Higher level of IL-10 and TNFα was also observed in HPV-positive women with different types of cervical lesions (20). In this study, we found that HeLa cells overexpressing LINC00673 showed increased IL-17A mRNA expression. IL-17 is a T-cell-derived cytokine that is thought to contribute to tumor growth and immune tolerance (21). Previous studies showed that IL-17A promotes cell proliferation in cervical cancer (22). Therefore, we speculate that high expression of LINC00673 in cervical cells may lead to the upregulation of IL-17A, and sustained IL-17A may promote the immune tolerance of mutant cells, leading to the occurrence of cervical cancer.

In this study, overexpression of LINC00673 increased the viability of HeLa and SiHa cells. Some studies showed that LINC00673 promotes proliferation in cervical cancer by activating the AKT signaling pathway (15). AKT, also known as PKB (protein kinase B), promotes cell proliferation by positively regulating the cell cycle (23). On the basis of the above studies, we hypothesized that LINC00673 regulates the activation of the AKT signaling pathway, leading to abnormal expression of genes associated with cell cycle and proliferation. Western blot analysis showed that the expressions of AKT, p-AKT, cyclin A2, and cyclin E were significantly increased in LINC00673-overexpressing cells. AKT plays a major role in promoting cell survival and resistance to apoptosis. When activated by AKT phosphorylation, MDM2 translocates to the nucleus and binds to the p53 tumor suppressor, promoting p53 degradation and thus promoting cell survival (24, 25). In this study, the protein levels of p53 and p21 were decreased in LINC00673-overexpressing cells. We also found that the epithelial-mesenchymal transition (EMT)–related proteins Snail and N-cadherin were increased in LINC00673-overexpressing cells. EMT is a process in which epithelial cells acquire the characteristics of mesenchymal cells, ultimately resulting in decreased intercellular adhesion and enhanced cell motility. The activation of AKT is also the core event of EMT (26, 27).

In summary, our results suggest that LINC00673 may serve as a useful target for the clinical diagnosis and treatment of cervical cancer. In addition, LINC00673 may represent a potential target for the treatment of cervical cancer and has broad application prospects. The specific mechanism of action of LINC00673 and its target genes and pathways still require further research.

## Data Availability Statement

The datasets presented in this study can be found in online repositories. The names of the repository/repositories and accession number(s) can be found in the article/**Supplementary Material**.

## Ethics Statement

The studies involving human participants were reviewed and approved by The ethical committee of Cancer Hospital, Chinese Academy of Medical Sciences. The patients/participants provided their written informed consent to participate in this study. The animal study was reviewed and approved by The animal ethical committee of Cancer Hospital, Chinese Academy of Medical Sciences.

## Author Contributions

All authors listed have made a substantial, direct, and intellectual contribution to the work and approved it for publication.

## Funding

This work was supported by the National Natural Science Foundation of China (Grant no.81872038) (Grant no.81902503), CAMS Innovation Fund for Medical Sciences (CIFMS) (Grant no.2016-I2M-1-001) and the Fundamental Research Funds for the Central Universities (Grant no. 3332019056).

## Conflict of Interest

The authors declare that the research was conducted in the absence of any commercial or financial relationships that could be construed as a potential conflict of interest.

## Supplementary Material

Supplementary Figure 1 Overexpression of LINC00673 in HeLa and SiHa cells. **(A, B)** Real-time PCR of LINC00673 expression in HeLa and SiHa cells. Data are presented as mean ± SD (N = 3); ****P* < 0.001. Student’s *t*-test.

Supplementary Figure 2 LINC00673 influences the expression of inflammatory cytokines. **(A, B)** RT-qPCR results showed that LINC00673 overexpression did not affect IL-6, IL-10 and Hsp90a mRNA expressions in HeLa **(A)** and SiHa **(B)** cells.
