# Supplementary figures and images for: Overexpression of LINC00673 Promotes the Proliferation of Cervical Cancer Cells
Source: Front Oncol. 2021 May 21;11:669739. doi: 10.3389/fonc.2021.669739 (PMC8176101; doi:10.3389/fonc.2021.669739)

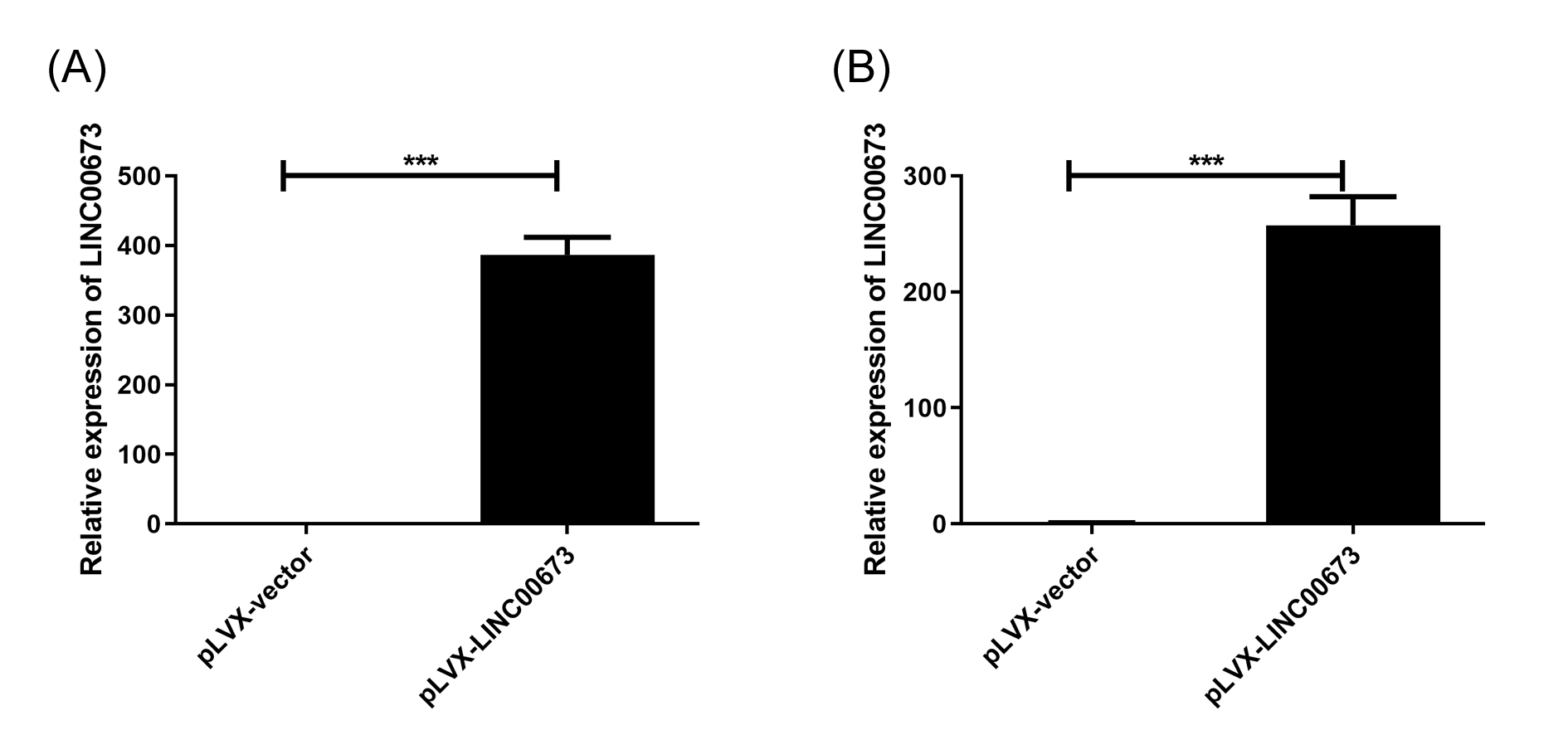

Supplement: Supplementary Figure 1 — Overexpression of LINC00673 in HeLa and SiHa cells. (A, B) Real-time PCR of LINC00673 expression in HeLa and SiHa cells. Data are presented as mean ± SD (N = 3); ***P < 0.001. Student’s t-test. [file Image_1.tif]

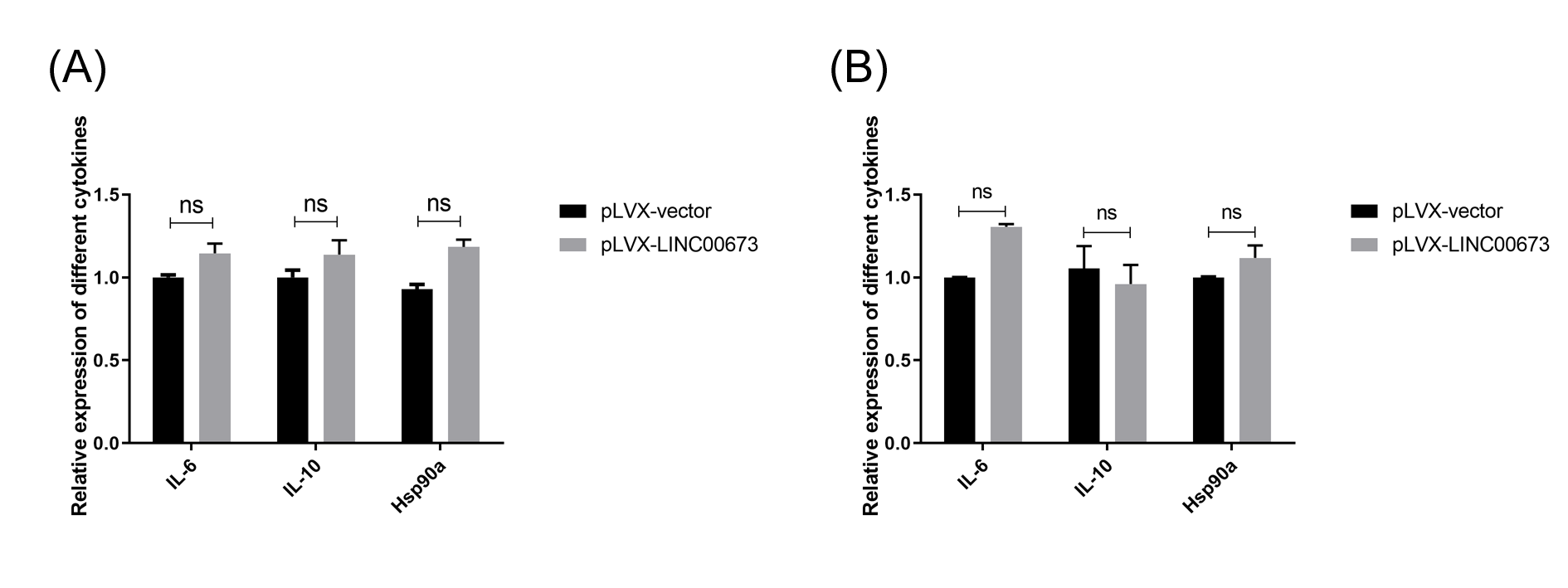

Supplement: Supplementary Figure 2 — LINC00673 influences the expression of inflammatory cytokines. (A, B) RT-qPCR results showed that LINC00673 overexpression did not affect IL-6, IL-10 and Hsp90a mRNA expressions in HeLa (A) and SiHa (B) cells. [file Image_2.tif]
