# Supplementary material for: Overexpression of LINC00673 Promotes the Proliferation of Cervical Cancer Cells
Source: Front Oncol. 2021 May 21;11:669739. doi: 10.3389/fonc.2021.669739 (PMC8176101; doi:10.3389/fonc.2021.669739)
Supplement: Supplementary file 3 [file DataSheet_1.docx]

**Table S1.** RT-qPCR primers

| **Official Symbol** | **Primer (5’-3’)** |
| --- | --- |
| LINC00673-F | CTGCTCTTTGGCCTTGGATG |
| LINC00673-R | ACGGATGGAGAAGAGGTCGT |
| Hsp90α-F | CTGAGAAGCAGGGCACCTGT |
| Hsp90α-R | GCAATATAAATGGCTGCAGATC |
| IL6-F | CCTGAACCTTCCAAAGATGGC |
| IL6-R | TTCACCAGGCAAGTCTCCTCA |
| IL10-F | GACTTTAAGGGTTACCTGGGTTG |
| IL10-R | TCACATGCGCCTTGATGTCTG |
| IL17A-F | TCCCACGAAATCCAGGATGC |
| IL17A-R | GGATGTTCAGGTTGACCATCAC |
| GAPDH-F | CCTGGTATGACAACGAATTTG |
| GAPDH-R | CAGTGAGGGTCTCTCTCTTCC |
